# Influence of Helium and Nitrogen as Quenching Atmospheres on the Amorphous Formation, Optimum Annealing Window, Soft Magnetic Properties, and Core Losses of Fe80B13Si7 Melt-Spun Ribbons

**DOI:** 10.3390/ma19173576

**Published:** 2026-08-23

**Authors:** K. M. Saiful Alam, Thomas Kresse, Roland Stein, Ralf Löffler, Gerhard Schneider, Dagmar Goll

**Affiliations:** Materials Research Institute, Aalen University, 73430 Aalen, Germany

**Keywords:** amorphous soft magnets, melt-spun ribbons, Fe80B13Si7, quenching atmospheres, soft magnetic properties, core loss

## Abstract

Fe-based amorphous soft magnets have long been investigated with respect to alloy design and processing strategies to enhance saturation polarization (*J_s_*) while preserving the amorphous phase essential for excellent soft magnetic properties and superior performance. This study demonstrates that Fe80B13Si7 melt-spun ribbons, containing a moderately high ferromagnetic fraction (~80 at%), display excellent amorphous stability, impressive soft magnetic behavior, and extremely low energy losses when processed under a highly efficient quenching atmosphere provided by helium. With the identical processing parameters, soft magnetic ribbons produced in helium gas provide a fully amorphous structure, whereas the ribbons synthesized in nitrogen undergo partial crystallization. The helium-quenched samples exhibit an exceptionally low mean coercivity (*H_c_*) of ~1.3 A/m, in contrast to the nitrogen-quenched ones (mean *H_c_*~14 A/m). The attained maximum permeability (*μ_max_*) in helium (28.4 ± 1.3 (×10^3^)) is even comparable with commercial Metglas 2605SA1 (33.3 ± 4.8 (×10^3^)). The average saturation polarization (*J_s_*) of the ribbons fabricated in both helium (~1.63 T) and nitrogen (~1.61 T) gases exceeds the commercial reference (~1.55 T). The helium environment showcases an excellent surface profile relative to nitrogen-induced quenching, which even shows a lower arithmetic mean surface height (*S_a_*) than the reference material. Furthermore, the optimum annealing window for minimizing coercivity is found to lie approximately 15 to 20 K below the Curie temperatures (*T_c_*) of the respective specimens. Core loss (*P_core_*) measurements reveal substantial loss reduction in helium-quenched ribbons relative to nitrogen-quenched ones and even slightly lower than Metglas 2605SA1 at a lower polarization level (*J*~0.5 T). Therefore, this work establishes a comprehensive understanding of how helium and nitrogen gases, as quenching environments, influence the amorphous formation, magnetic softness, surface morphology, and energy losses of an alloy with a relatively high Fe content from the Fe-B-Si family to harness the material’s maximum potential.

## 1. Introduction

Soft magnetic materials (SMMs) are substances that can easily be magnetized or demagnetized. They are widely used in applications such as power generation, energy conversion and transport, power electronics, sensors, etc. [1,2,3,4]. Power-distributing transformers are integral components of electrical systems for efficient energy transmission, and the selection of core material directly impacts their performance and efficiency. Grain-oriented silicon steel is the most commonly used soft magnetic material (SMM) for transformer cores due to its high permeability and low core loss [5]. Even with this material, energy losses persist, which inherently limit the maximum attainable efficiency. Reducing these losses is critical, as even small improvements can yield substantial energy savings. However, modern-day transformers constructed with grain-oriented electrical steel have reached a level of technical advancement where very little space is left for loss minimization. On the contrary, with the advent of the melt-spinning method, continuous amorphous soft magnetic ribbons can be fabricated through rapid quenching. These amorphous soft magnets (ASMs) exhibit excellent magnetic softness and extremely low core losses, making them promising materials for power-distribution transformers and other inductive components in electrical devices [6,7].

ASMs are typically prepared by incorporating approximately 20% metalloids/alloying elements, such as Si, B, Al, C, and P, into Fe-based and Co-based alloys [8]. Si and B are key metalloids that enhance glass-forming ability (GFA) and preserve the stability of the amorphous phase [9,10,11]. At the same time, Co-based systems are appropriate for high permeability [12], and Fe-based systems are applicable for high saturation polarization [13]. Following years of advancement, Fe-based amorphous alloys gained a competitive advantage in applications such as distribution transformers, intermediate-frequency transformers, and filter inductors due to their higher saturation polarization (*J_s_*) compared to Fe-Ni-based and Co-based amorphous alloys [14,15]. Although melt-spinning of Fe-based ASMs has been extensively explored, Fe-based amorphous alloys typically exhibit lower *J_s_* than traditional silicon steels, which can constrain their effectiveness in high-power-density applications [9]. Increasing the concentration of ferromagnetic elements to enhance the magnetic moment can negatively impact the alloy’s GFA, thereby complicating the manufacturing process [15]. Therefore, additional research is still required on alloy composition and processing strategies to obtain higher *J_s_* while conserving the amorphous phase. As a result, intensive investigation remains necessary to develop strategies to overcome persistent limitations and exploit the optimum functionality of these materials.

Fe-B-Si alloys are promising for technological applications due to their soft ferromagnetic characteristics, high GFA, and mechanical resilience [16,17,18]. Strong Fe-Si and Fe-B bonding, along with negative enthalpies of mixing, contribute to enhanced amorphous-forming ability (AFA) [19]. A fully amorphous structure is usually achieved within 5–26 at% B and 0–29 at% Si compositions [19]. B enhances glass-forming ability roughly five times more than Si [20], and has a less detrimental effect on *J_s_* than silicon [21,22]. However, it also reduces the temperature gap between crystallization peaks, increasing Fe–B compound formation, causing magnetic hardening, and eventually degrading soft magnetic properties [23,24,25]. Si-containing amorphous alloys from the Fe-B-Si system with roughly 80–82% iron are preferred because they offer maximum saturation polarization together with very low coercivity (*H_c_*), resulting in reduced losses as well as better manufacturability and stability [18]. Thus, the novelty of this proposed investigation stems from exploring processing-atmosphere engineering as an alternative route to optimize the structural and functional performance of high-Fe-containing Fe–B–Si amorphous alloys, using Fe80B13Si7 (at%) as a model composition. This alloy has shown attractive glass-forming potential and possesses an elemental balance conducive to achieving high *J_s_*. According to Li et al. [26] and Guan et al. [27], the Fe80B13Si7 metallic glass exhibited strong resistance to crystallization under high-intensity pulsed ion or He-ion beam exposure. Lung et al. [28] further showed that substituting C with Si in Fe80B13C7 enhanced the crystallization activation energy. Although the alloy showed promising GFA, a detailed assessment of its magnetic behavior and functional efficiency remains lacking.

Maintaining a fully amorphous structure and enhancing magnetic effectiveness require not only an optimized elemental balance but also processing conditions tailored for superior properties and performance. The surrounding gas used during rapid quenching strongly influences the surface quality and microstructures of melt-spun ribbons, and these characteristics significantly impact their soft magnetic properties [29,30,31,32,33]. Peng et al. [32] found that Fe84.8Zr3.4Nb3.4B7.4Cu1 nanocrystalline ribbons melt-spun in N_2_ exhibited pronounced microstructural texture, reduced squareness ratios (*J_r_*/*J_s_*), lower permeability, decreased amorphous-phase stability, and higher relaxation frequencies compared with those spun in Ar. Wang et al. [33] demonstrated that gas pressure influenced the microstructure and magnetic behavior of melt-spun Nd10Fe83Zr1B6 nanocomposites, with reduced pressure minimizing gas pocket formation, yielding a finer and more uniform microstructure. Consistent with this, Yapp et al. [34] also observed the absence of gas pockets and stable properties in NdFeB-based nanocrystalline ribbons processed under reduced pressure. In contrast to nitrogen (~0.026 W/m·K) and argon (~0.017 W/m·K), helium exhibits a notably higher thermal conductivity of ~0.15 W/m·K at room temperature and atmospheric pressure [35,36,37]. Such anomalies can directly affect the cooling rates, thereby affecting microstructural development and surface quality during material processing. Atraszkiewicz et al. [38] reported a defect-free surface layer microstructure and reduced mechanical distortion in toothed gears quenched under high-pressure helium compared with nitrogen. In laser powder bed fusion of 316L stainless steel, the use of helium enhanced melt-pool stability, lowered defect formation, and refined microstructural features, ultimately improving the mechanical performance relative to nitrogen and argon [39]. Furthermore, helium’s high cooling efficiency promotes rapid heat extraction and uniform temperature distribution in the metallic material, yielding more homogeneous structures, whereas slower nitrogen cooling can generate thermal gradients and may induce undesirable reactions that alter the microstructure and properties [40]. However, studies on the effects of ambient gases on the magnetic properties and energy efficiency of the melt-spun ribbons are still lacking. Unlike previous studies that mainly examined the effects of ambient gases on microstructure and surface defects in other alloy systems, this study focuses on a systematic investigation of the influence of processing atmospheres on amorphous phase formation, surface morphology, soft magnetic properties, and core loss behavior of a high-Fe-containing promising soft magnetic alloy. Thus, the novelty of this work lies in demonstrating how helium and nitrogen as quenching atmospheres can be implemented to tailor the structural quality and magnetic performance of Fe80B13Si7 melt-spun ribbons.

## 2. Materials and Methods

### 2.1. Sample Preparation and Reference Material

A three-component ingot (100 ± 10 g final mass after segmentation and grinding) with a nominal atomic composition of Fe80B13Si7 was synthesized from industrial-grade Fe (99.9 wt%), Si (99.9 wt%), and Fe-B (18.9 wt%) pre-alloy using an Indutherm VTC 200 VTi (Indutherm Heißsanlagen GmbH, Walzbachtal, Germany) high-performance vacuum tilt induction casting device. The reported alloy composition represents a nominal value based on the raw material weights, with an instrumental measurement error of ±0.5 mg. Discotom-100 precision cut-off machine (Struers GmbH, Willich, Germany) was used to section the main ingot into multiple pieces of 15–20 g, ensuring all subsequent melt-spinning runs originated from a single batch to minimize compositional variation and external influence. Rapidly quenched ribbons, approximately 9 mm in width, were produced from the pre-alloy ingots in controlled helium and nitrogen atmospheres using a high-vacuum single-copper-roller melt-spinning device (Melt Spinner HV from Edmund Bühler GmbH, Bodelshausen, Germany). Additionally, Metglas^®^ 2605SA1, a commercial amorphous alloy ribbon developed by Metglas Inc., Conway, SC, USA, was chosen as a reference material, which is widely known for its extremely low iron losses and excellent soft magnetic properties despite having a different chemical composition and processing technique compared to the investigated material. Table 1 summarizes the key process variables and physical characteristics of both the in-house synthesized ribbons and the reference material.

**Figure 1:**
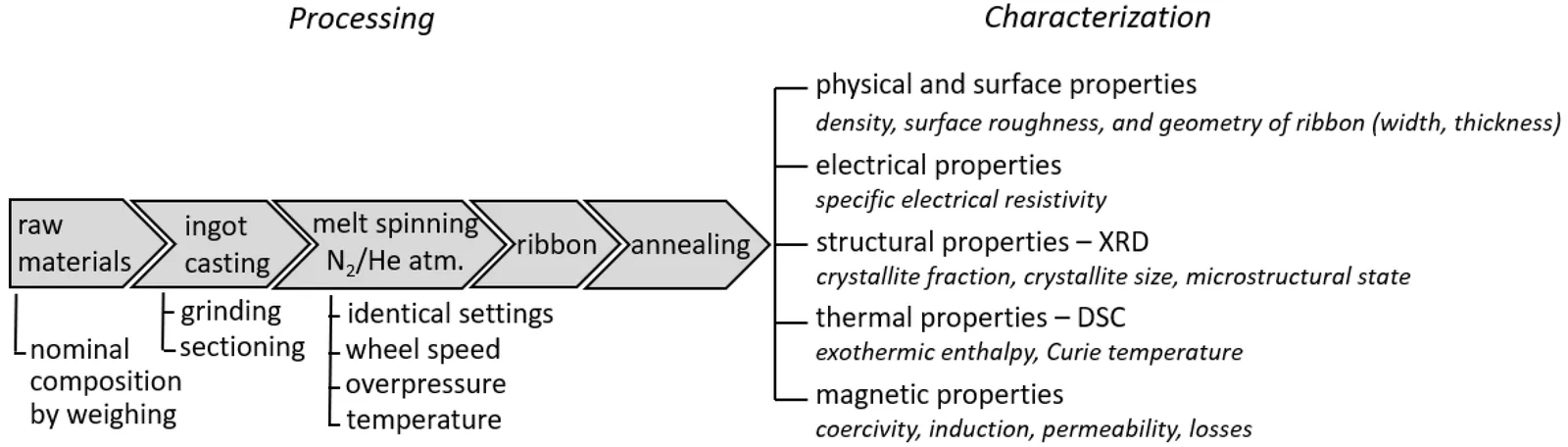
Processing steps of the in-house fabricated Fe80B13Si7 melt-spun ribbons and the reference material.

For ease of interpretation, ribbons produced under helium and nitrogen are denoted as MS-He and MS-N_2_, respectively. A wide range of process parameters were implemented in both nitrogen and helium to produce continuous melt-spun Fe80B13Si7 ribbons with sufficient mechanical integrity and high ribbon yield. The wheel velocity and chamber overpressure were varied during preliminary experiments, while the melt-ejection temperature was maintained at approximately 1240 °C (~50 °C above the onset of melting). For each processing condition, the resulting ribbon continuity, yield length, mechanical flexibility, and tendency toward fracture were evaluated. According to these preliminary trials, the optimum process variables shown in Table 1 provided the best formability (180° bending/folding without any sign of fractures) and maximum ribbon yield in terms of length. Under other processing conditions, one or more constraints, including increased fragility, inconsistent ribbon width, higher porosity, or substantially low ribbon yield, were observed, which restrict their processability and effectiveness in further characterization and comparative analysis. Moreover, several trials were conducted for the preferred process variables to examine their reproducibility in both nitrogen and helium, yielding similar dimensions (thickness and width) and structures (XRD pattern), along with comparable coercivity. Magnetic, thermal, and physical properties were analyzed on one continuous ribbon from MS-N_2_ and MS-He along the length. Hence, data acquired from the characterization of MS-He and MS-N_2_ have been illustrated in this study as representatives of the respective process variables shown in Table 1. Furthermore, processing steps of the melt-spun ribbons are depicted in Figure 1.

### 2.2. Thermal Behavior and Heat Treatment

The DSC 404 F3 Pegasus^®^ from NETZSCH-Gerätebau GmbH, Selb, Germany, performed differential scanning calorimetry (DSC) analysis at a heating rate of 0.167 K/sec under an argon atmosphere up to 650 °C, providing insights into the thermal behavior of the samples. The change in enthalpy for crystallization was calculated by integrating the area under the corresponding exothermic peak and dividing by the heating rate, according to the ISO11357-1 standard [43], using the following equation:

(1) $$\Delta H_{\mathrm{cryst}}\;=\;\int_{T1}^{T2}\frac{q(T)}{\beta }\;dT$$

where:

Δ*H_cryst_* = change in enthalpy for crystallization in J/g;

*q*(*T*) = heat flow rate per unit mass in mW/mg, as a function of temperature (*T*);

*T*_1_ and *T*_2_ = integration limits for the corresponding crystallization peak;

*β* = heating rate in °C/s.

Stress-relief annealing heat treatment (HT) of the ribbons was performed in air at varying temperatures for 15 min using a drying oven from Nabertherm GmbH, Lilienthal, Germany. In-house produced ribbons were annealed from 360 °C to 420 °C with intervals of 15 K to find the appropriate annealing temperature for minimum coercivity, while Metglas 2605SA1 was annealed at 400 °C based on the literature data [44]. Additionally, the Curie temperature (*T_C_*) was determined from the DSC heating curves using the NETZSCH Proteus Thermal analysis software (version 7.x). *T_C_* was extracted as the onset temperature of the transition causing a subtle heat-flow deviation in the DSC curve appearing before the first crystallization onset.

### 2.3. Characterization of Structural, Physical, and Magnetic Properties

The mean density and thickness were measured using the Archimedean principle. X-ray diffraction (XRD) measurements were carried out to analyze the crystallization tendency of the ribbons over a 2-θ range of 30 to 100° with Co-Kα radiation using the Seifert Sun XRD X-ray diffractometer from DSeTec GmbH, Munich, Germany. The crystallite sizes and weighted amorphous fractions of the samples were estimated based on the work of Dos Santos et al. [45]. The amorphous fraction (*X_am_*) was quantified using the integrated areas of the amorphous hump (*A_am_*) and crystalline peaks (*A_cr_*) extracted via RayFlex Analyze software (version 2.503) according to *X_am_* = [*A_am_*/(*A_cr_* + *A_am_*)] × 100%. The apparent crystal size (*D*) was calculated using the Scherrer formula (*D* = 0.94 *λ_CoKa1_*/(FWHM × cosθ)) for wavelength *λ_CoKa1_*~0.1789 nm. The FWHM values and peak positions were determined by RayflexAnalyze software.

Magnetic properties and efficiency were measured using a range of specialized equipment. The Brockhaus MPG 200 D tester (Dr. Brockhaus Messtechnik GmbH, Lüdenscheid, Germany) enables DC and AC field analysis of ultra-thin foils. Quasi-static magnetic hysteresis loops were recorded under applied fields ranging from −5000 A/m to 5000 A/m at a sweep rate of 50 mT/s. Core loss (*P_core_*) evaluations were performed at frequencies between 50 Hz and 400 Hz with polarization (*J*) amplitudes set at 0.5 T and 1.0 T. Maximum permeability (*μ_max_*) was measured using the same device in DC commutation (DCK) mode, with a field sweep rate of 50 mT/s and a maximum applied field of 5000 A/m. Magnetic coercivity (*H_c_*) was determined with the Förster Koerzimat 1.097 HCJ (Dr. Foerster GmbH, Reutlingen, Germany), and saturation polarization (*J_s_*) was quantified using a Quantum Design PPMS-9T vibrating sample magnetometer (Quantum Design, Inc., San Diego, CA, USA). The specific electrical resistivity (*ρ*) measurements of the ribbons were conducted at room temperature using the RT-70V four-point probe system from Napson Corporation, Tokyo, Japan. The average of ten measurements was used to obtain higher accuracy. The surface topography was examined using a high-precision, non-contact 3D surface profiler named Zegage™ White Light Interferometer from Zygo Corporation, Middlefield, CT, USA, equipped with a 10x objective. For each sample, five distinct regions on the surface were examined to reduce measurement variability.

Analyses were generally conducted on samples extracted from the leading edge, midpoint, and trailing edge of the continuous as-spun ribbon to account for potential inhomogeneities along the ribbon length. The dimensions were tailored to meet the requirements of the respective measurement techniques. Ten independent readings were obtained for *H_c_* and *ρ* measurements of each sample, five for *S_a_*, and three measurements were performed for *P_core_* and *μ_max_* to assess potential deviations. The standard deviations in different properties (e.g., *H_c_*, *P_core_*, *μ_max_*, *S_a_*, and *ρ*) reflect the variability along the length of the ribbons MS-N_2_ and MS-He.

## 3. Results

### 3.1. Amorphous Phase Formation

**Figure 2:**
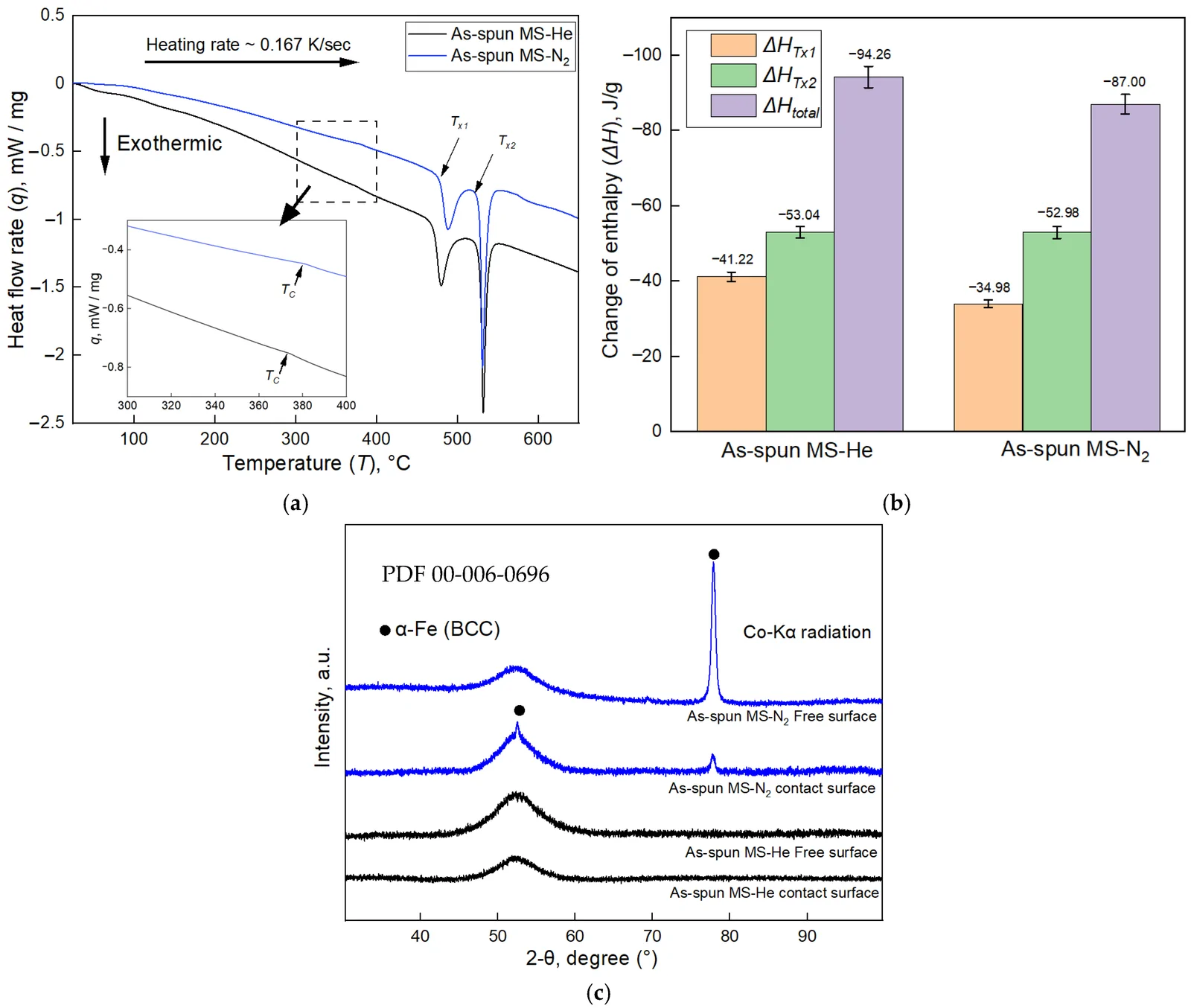
(**a**) DSC curves, (**b**) corresponding change in enthalpies for crystallization, and (**c**) X-ray diffractograms of the as-quenched ribbons.

The thermal properties and crystallization tendency of the samples fabricated in helium and nitrogen environments are shown in Figure 2. Figure 2a, Figure 2b, and Figure 2c denote the DSC curves, crystallization enthalpies, and X-ray diffractograms of the as-spun ribbons, respectively. Distinct exothermic peaks corresponding to the first (α-Fe precipitates) and second (Fe-metalloid compounds, e.g., Fe-B) crystallization events are clearly observed for both MS-He and MS-N_2_, as shown in Figure 2a. In terms of the primary (*T*_*x*1_) and secondary (*T*_*x*2_) crystallization onset temperatures, minimal differences can be observed between the as-quenched ribbons produced in nitrogen (*T*_*x*1_-475 ± 5 °C and *T*_*x*2_~522 ± 5 °C) and helium (*T*_*x*1_-470 ± 5 °C and *T*_*x*2_-521 ± 5 °C). Similarly, the Curie temperatures (*T_c_*) show minor deviation between MS-He (~375 °C) and MS-N_2_ (~381 °C). However, Figure 2b shows larger differences between MS-He and MS-N_2_ in the enthalpy changes for primary (Δ*H*_*Tx*1_) and total crystallization (Δ*H_total_*) than for secondary crystallization (Δ*H*_*Tx*2_). Since the base material is identical for both samples, the higher Δ*H_total_* of MS-He is consistent with a lower initial degree of crystallinity than that of MS-N_2_. In other words, the larger retained amorphous fraction in MS-He is associated with a larger exothermic enthalpy release during crystallization from the as-quenched state. However, effects arising from differences in crystallization kinetics between the samples cannot be excluded. This phenomenon can also be confirmed from the XRD patterns of the as-spun ribbons, as exhibited in Figure 2c. A broad amorphous halo between 47° and 57° angles can be observed for both samples on the contact and free surfaces. The MS-He sample in the as-quenched state shows fully amorphous diffractograms on both sides, while as-spun MS-N_2_ undergoes partial crystallization, as evidenced by ferromagnetic α-Fe (BCC) peaks on both sides. On the free and contact sides, a large and a small peak are observed near the 2-θ angle of ~78°, respectively, with a minor peak overlaying the amorphous halo on the contact surface of MS-N_2_. Estimated mean crystallite sizes on the contact and free surfaces correspond to ~22.8 nm and ~20.2 nm, with weighted amorphous fractions of ~92% and ~66%, respectively. This variation denotes an inhomogeneous crystal distribution across the thickness of MS-N_2_. Hence, a higher tendency of crystallization can be observed from the DSC and XRD results in the as-spun samples produced in nitrogen.

### 3.2. Effect of Annealing Temperature on the Coercivity

**Figure 3:**
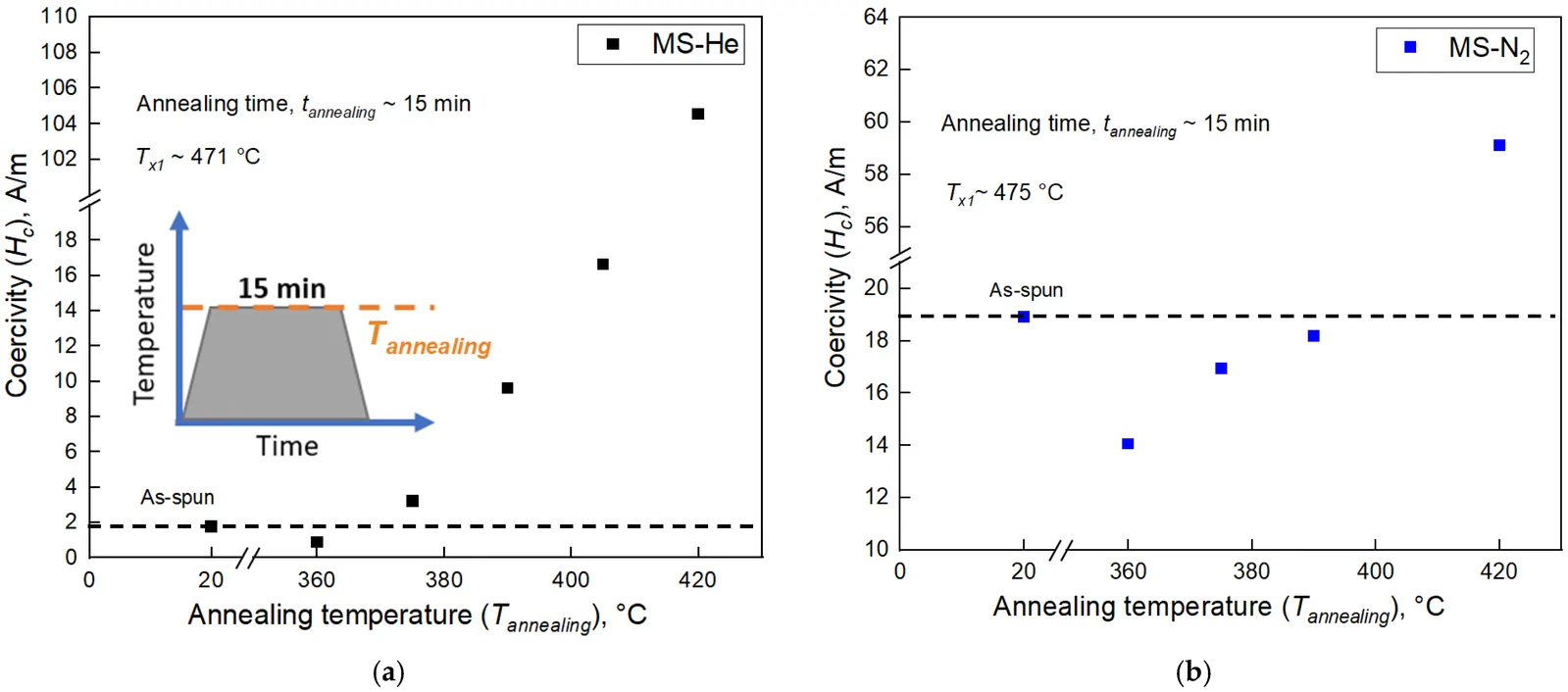
Effect of annealing temperature on the coercivity of Fe80B13Si7 melt-spun ribbons produced in (**a**) helium and (**b**) nitrogen (the regions falling under the horizontal lines denote optimum windows for annealing).

Melt-spun ribbons are commonly annealed to relieve internal stresses induced by rapid quenching, which in turn enhances magnetic properties such as coercivity [46,47,48]. Selecting an appropriate annealing temperature is critical for soft magnetic ribbons, as heat-induced microstructural changes can undesirably alter their properties [44,46,47]. Accordingly, in Figure 3a and Figure 3b, the influence of annealing temperature on the coercivity of MS-He and MS-N_2_ is presented, respectively. Minimum average coercive fields are found at analogous annealing points (~360 °C) for both, regardless of their quenching atmosphere. After the minimum coercivity point is achieved, the mean coercive force tends to increase with annealing temperature for both MS-He and MS-N_2_. The following properties (e.g., *J_s_*, *μ_max_*, *ρ*, and *P_core_*) of the heat-treated samples in the current study are measured at the optimum annealing point for minimum *H_c_*.

### 3.3. Key Magnetic and Physical Properties

In Table 2, magnetic coercivity, maximum permeability, specific electrical resistivity, surface profile, and saturation polarization of the MS-He, MS-N_2_, and reference material are illustrated. *H_c_* tends to decrease, whereas *μ_max_* tends to increase after annealing. Nevertheless, the changes in resistivities are negligible after the heat treatment for the corresponding samples. Both in the as-quenched and annealed states, MS-He depicts significantly lower *H_c_* than MS-N_2_. However, MS-He displays a lower coercive field than the reference in the as-quenched state, which slightly surpasses the reference material after annealing. MS-He exhibits the highest *μ_max_* among the three samples in the as-fabricated state, while after heat treatment, MS-He and Metglas 2605SA1 show minimal difference, which is substantially larger than that of MS-N_2_. Specific electrical resistivities (*ρ*) of all the samples are comparable in both as-spun and heat-treated conditions. The saturation polarizations of both MS-He and MS-N_2_ are comparable, with values clearly higher than the benchmark sample (Table 2).

**Figure 4:**
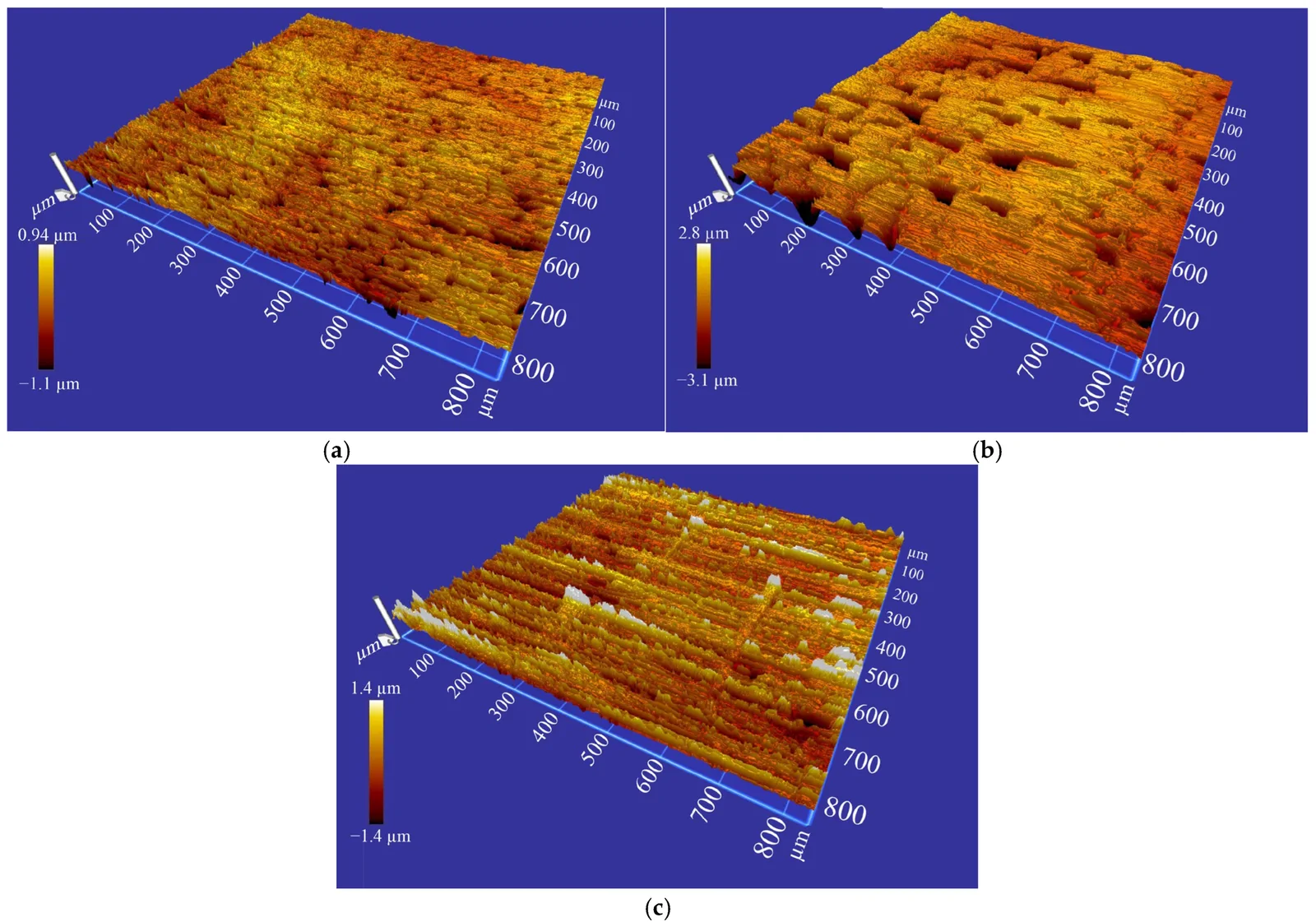
Color-coded 3-D surface profiles of as-spun (**a**) MS-He, (**b**) MS-N_2_, and (**c**) Metglas 2605SA1.

MS-He has shown the minimum arithmetic mean height of the surface (*S_a_*) among the samples, while MS-N_2_ reflects approximately two times higher amplitude. Metglas 2605SA1 exhibits a slightly larger areal arithmetic mean roughness than MS-He. Nevertheless, from the color-coded topography maps of the corresponding samples, as demonstrated in Figure 4, it is clearly visible that MS-N_2_ exhibits higher undulations in its surface morphology, ranging from −3.1 to 2.8 µm, unlike the other two samples.

### 3.4. Core Losses and Quasi-Static Hysteresis Loops

**Figure 5:**
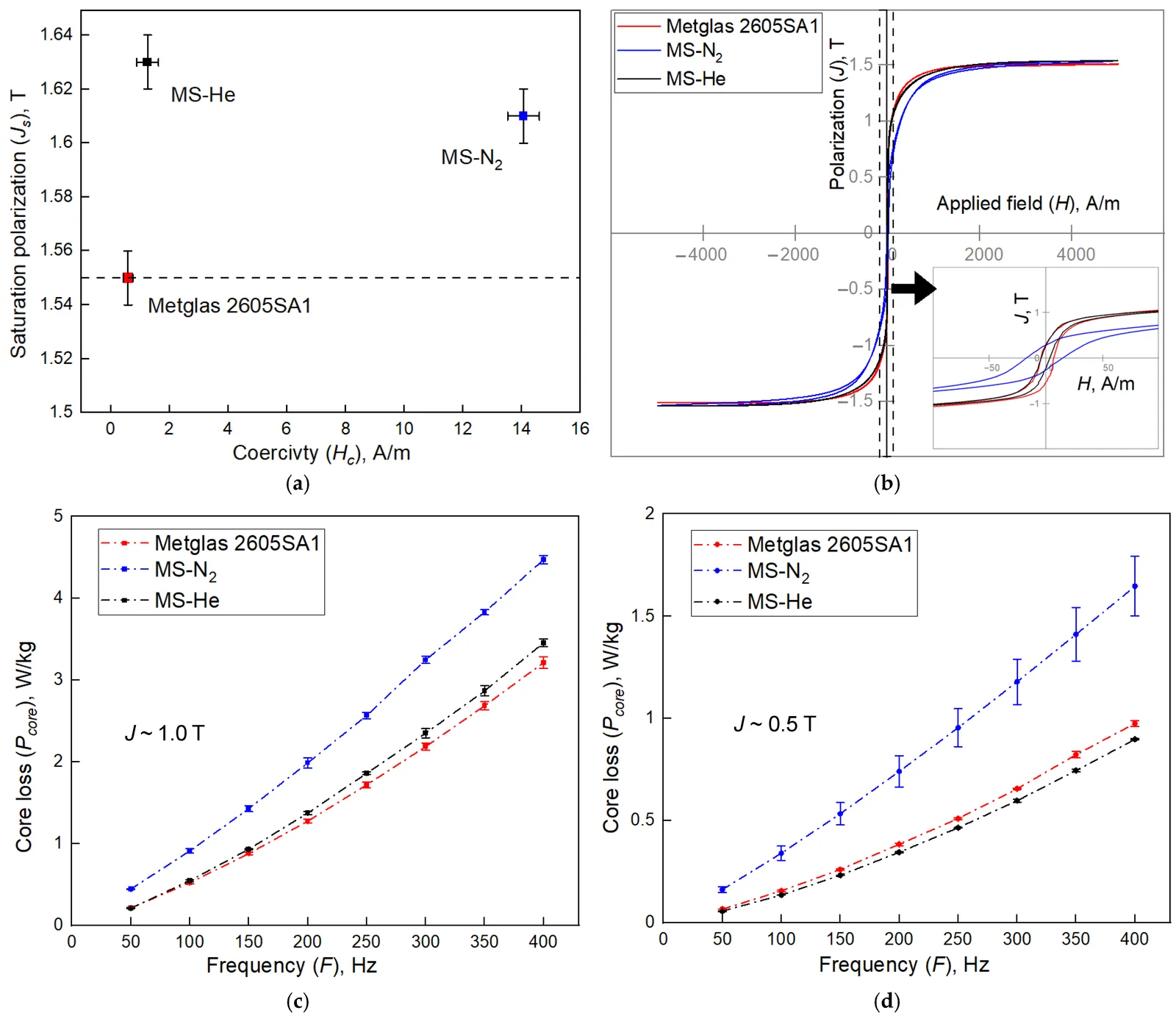
(**a**) *H_c_* vs. *J_s_*, (**b**) quasi-static hysteresis loops, (**c**) *F* vs. *P_core_* at *J*~1.0 T, and (**d**) *F* vs. *P_core_* at *J*~0.5 T of the annealed samples.

Figure 5a, Figure 5b, Figure 5c, and Figure 5d represent the *H_c_* vs. *J_s_*, quasi-static hysteresis loops, *F* vs. *P_core_* at 1.0 T and 0.5 T, respectively, for the corresponding samples in their annealed states. MS-He and MS-N_2_ both show comparable *J_s_*, which exceed the value obtained from commercial Metglas 2605SA1. Melt-spun Fe80B13Si7 ribbons produced in helium exhibit *H_c_* values in both the as-spun and annealed states that are an order of magnitude lower than those of samples produced in nitrogen. However, the mean coercive force (~1.3 A/m) of MS-He is only slightly higher than that of the commercial benchmark (~0.6 A/m), which is still extremely low. From the quasi-static hysteresis loops of the heat-treated samples shown in Figure 5b, it can be observed that MS-N_2_ represents a wider loop at low fields compared with MS-He and the reference material, showcasing narrower loop widths. However, the quasi-static *J-H* loops of MS-He and the reference material show an analogy with each other at a low applied field of *H* < 100 A/m (inset of Figure 5b). The core losses of MS-N_2_ for varying frequency at 1.0 T and 0.5 T of polarization, as shown in Figure 5c and Figure 5d, respectively, are significantly higher than the total energy losses of MS-He and Metglas 2605SA1. The differences in the losses of MS-He and the reference are minimal in the low-frequency range from 50 to 100 Hz at both lower and higher polarizations. However, at 0.5 T amplitude, the losses in MS-He are lower than Metglas 2605SA1 in a comparatively higher frequency range, whereas an opposite trend is observed for 1.0 T of polarization.

## 4. Discussion

### 4.1. Amorphous Phase Formation

Based on DSC and XRD results, identical processing parameters under a helium atmosphere produced a fully vitrified structure. In contrast, the nitrogen-quenched samples showed partial crystallization and an increased propensity towards devitrification. This behavior reflects the enhanced formability of the amorphous phase in helium. The underlying mechanism is attributed to helium’s significantly greater thermal conductivity, approximately six times larger than that of nitrogen [35,36,37], which is expected to have improved heat dissipation from the melt and may have consequently increased the effective cooling rate required to facilitate glass formation. Furthermore, the slow heat extraction in the nitrogen atmosphere is likely to have induced the variability of crystallinity across the ribbon thickness of MS-N_2_.

### 4.2. Effect of Annealing Temperature on Coercivity

At annealing temperatures slightly below *T_c_*, amorphous ribbons can still develop favorable soft magnetic characteristics [49,50]. Therefore, the initial annealing temperature was set to 15 to 20 °C below the Curie temperatures of MS-He and MS-N_2_, which was then gradually raised to 420 °C with intervals of 15 K. Consequently, both the amorphous and partially amorphous Fe80B13Si7 melt-spun ribbons reached their minimum coercivity at the same annealing temperature (~360 °C), slightly below their corresponding Curie temperatures, but with notably distinct minimum coercivity values. The event of similarity in optimal annealing temperatures arises because the crystallization kinetics or microstructural change in the ribbons depend heavily on composition and thermal activation, setting the onset and growth conditions for microstructural evolution, irrespective of initial structure or processing atmosphere [51,52,53]. Hence, MS-He and MS-N_2_ obtained the desired minimum *H_c_* at approximately 360 °C.

### 4.3. Key Magnetic and Physical Properties

**Figure 6:**
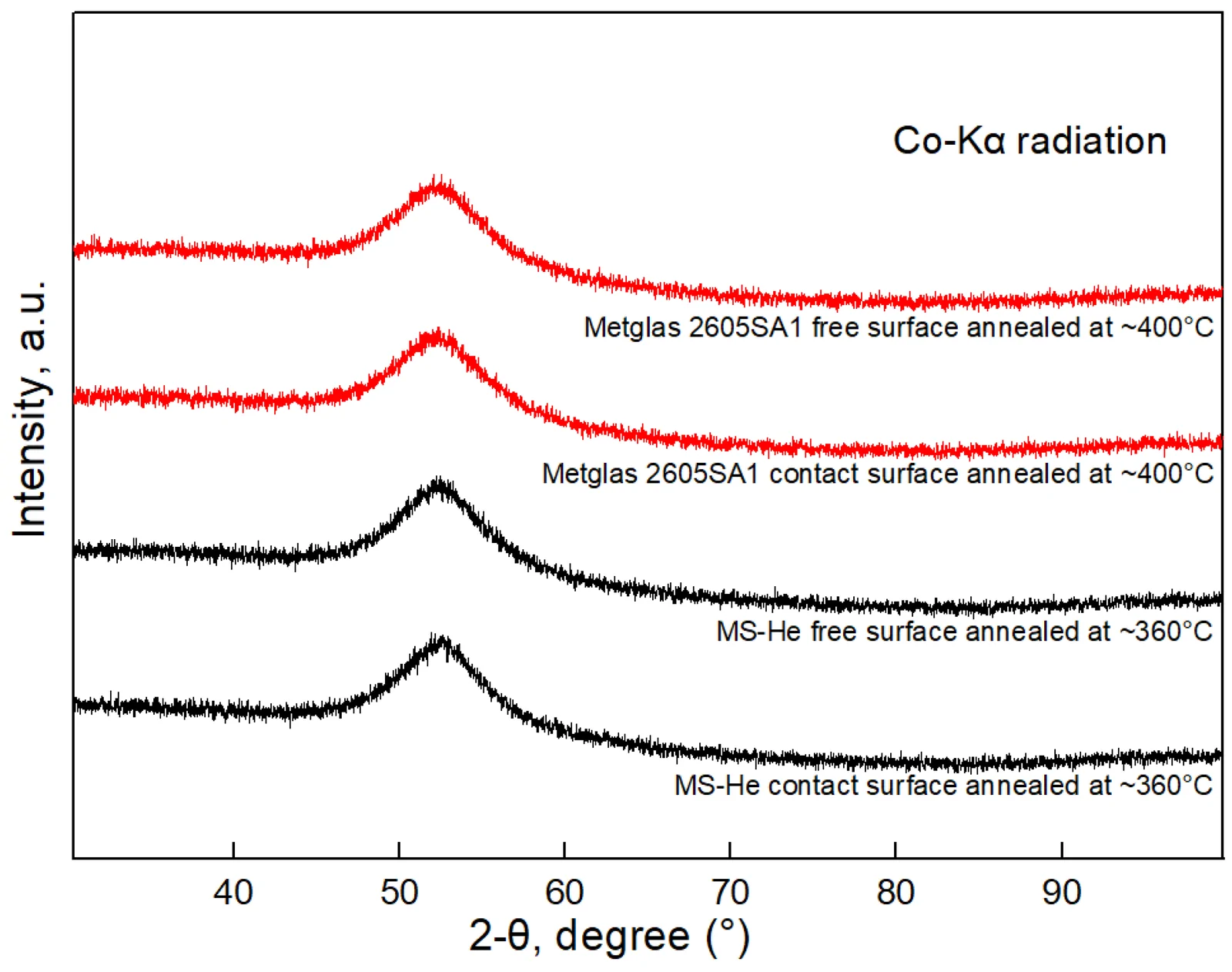
X-ray diffractograms of MS-He and Metglas 2605SA1 annealed at 360 °C and 400 °C, respectively.

In both as-spun and heat-treated conditions, MS-He and Metglas 2605SA1 exhibited significantly lower mean *H_c_* than MS-N_2_. In amorphous materials, the structural correlation length (*D*) is generally much smaller than the exchange length (*L*_0_), even smaller than that of nanocrystalline soft magnets, resulting in extremely low anisotropy under the random-anisotropy model and yielding very low coercivity and excellent magnetic softness [9]. Coupled with this, the lack of crystallinity in MS-He and the commercial reference results in low anisotropic energy, contributing to the reduced coercivity. However, the coercivity of nanocrystalline MS-N_2_ is on the higher side than expected based on its estimated grain size according to the relationship between the coercivity and grain size for various soft magnetic materials shown by Herzer [9]. This may be attributed to structural inhomogeneities across the thickness of MS-N_2_ that can impede magnetic domain wall movement and shift the material toward higher coercivity. During rapid solidification from liquid to solid, Fe atoms are immobilized, inducing high internal stresses [54,55,56]. In addition, pronounced temperature gradients result in residual stresses [57]. These stresses can act as pinning factors for the domain wall motion during magnetization in the as-spun state, leading to low maximum permeability in the samples, as shown in Table 2. The heat treatment was performed well below *T*_*x*1_ for all ribbons, primarily relieving internal stresses without inducing crystallization in the amorphous ribbons (Figure 6). This stress relief allows domain walls to move more freely during magnetization, significantly increasing maximum permeability in the amorphous MS-He and Metglas 2605SA1. In MS-N_2_, crystallite shape anisotropy and the coexistence of pre-existing grains can restrict the benefits of stress relaxation by hindering domain wall mobility, thereby limiting permeability gains after isothermal annealing [9,58,59,60]. The specific electrical resistivity (*ρ*) of in-house fabricated melt-spun ribbons and Metglas 2605SA1 is comparable in both as-spun and heat-treated states. This behavior is consistent with data and discussion for Fe-B-Si-based amorphous/nanocrystalline ribbons and Metglas 2605SA1, which roughly range from 130 to 180 µΩ·cm [42,61]. The disordered structures of amorphous materials directly hinder electron mobility, leading to relatively high *ρ* compared to the conventional polycrystalline counterparts, while partially amorphous materials with embedded nanocrystals in an amorphous matrix scatter electrons by the potential barriers of grain/matrix interfaces, resulting in a resistance on the higher side [62,63]. Since the heat treatments of the samples were executed well below the *T_X1_* for relieving internal stresses, it is more likely to have modified magnetic properties without altering microstructures and the electron-transporting environment. Hence, the deviations in resistance before and after annealing are insignificant.

Helium has a much higher thermal conductivity and lower density than nitrogen, so convective heat transfer from the melt into the gas is stronger and more uniform in He, which promotes faster and more homogeneous solidification of the surface and reduces surface waviness [35,36,37,40] as observed on MS-He. Additionally, helium enhances the stability of the melt puddle and lowers defect formation, contributing to an improved surface quality and reduced mechanical distortion [38,39]. Hence, MS-He provided an excellent surface profile compared to MS-N_2_ and eventually showed reduced surface undulations compared to Metglas 2605SA1. Although crystallinity and residual stresses from quenching are likely the dominant factors, surface morphology is one of the pinning factors that can also affect *H_c_* [64]. Stress-induced magnetic anisotropy arising from surface irregularities acts as domain-wall pinning centers, thereby influencing domain-wall motion in melt-spun ribbons [65,66]. Conversely, a smoother surface morphology can result in enhanced soft magnetic properties [67]. Consequently, improved surface finish and structural uniformity reduce magnetic anisotropy, resulting in a lower *H_c_* in Fe-based amorphous ribbons [67,68]. Therefore, the lower arithmetic mean height of the surface of MS-He may have partially contributed to its extremely small mean coercivity in contrast to MS-N_2_.

Saturation polarization is generally considered an intrinsic property primarily controlled by alloy composition and the ratio of magnetic to non-magnetic phases. In this study, *J_s_* was measured for the samples in their annealed states. The minor deviations between as-quenched and heat-treated states were disregarded, as the heat treatments were performed well below *T_X1_* and therefore were not expected to noticeably modify the microstructures (e.g., size or fraction of ferromagnetic α-Fe crystallites) and *J_s_*. MS-He and MS-N_2_ exhibited nearly identical saturation polarization (Figure 5a and Table 2) because they originated from the same alloy composition (Fe80B13Si7). In contrast, the reference material with an alloy composition of Fe80B11Si9 showcased a value on the lower side. The underlying reason behind this difference can be explained by the proportions of metalloid content, as B and Si contribute differently to magnetic properties. Based on an empirical formula for Fe-based amorphous and nanocrystalline alloys, the magnetic atomic valences of Fe, B, and Si are 2, −3, and −4, respectively [17,18]. Since an atom’s average magnetic moment is proportional to its average magnetic atomic valence, Si has a more adverse effect on *J_s_* than B [17]. As a result, the higher Si content in Metglas 2605SA1 led to a reduced saturation polarization, despite having a similar ferromagnetic fraction relative to MS-He and MS-N_2_.

### 4.4. Core Losses and Quasi-Static Hysteresis Loops

In magnetic core loss analysis, the commonly applied approach is the separation of losses principle [1,69,70]. Under this framework, the total iron or core loss is expressed as the sum of hysteresis loss (*P_hys_*) and dynamic loss (*P_dyn_*). The dynamic part itself consists of classical eddy current loss (*P_eddy_*) and excess loss (*P_exc_*). In this study, losses are only shown for annealed samples, as the most favorable soft magnetic properties (e.g., *H_c_*, *μ_max_*) were obtained in heat-treated conditions. The measured samples were single-layer ultra-thin foils with comparable electrical resistivity. Moreover, for thin Fe-based amorphous or nanocrystalline ribbons, the eddy current losses are so small that they are disregarded, and in the investigated low-frequency range (50–400 Hz), the hysteresis losses are the dominant part; the rest are excess losses [1,71]. To further understand the loss mechanism, contributions of different loss components were quantified from the total losses of the corresponding samples using Bertotti’s loss separation model [72] as described by Hilzinger and Rodewald [73]. These total losses were separated by analyzing a *F* vs. *P_core_*/*F* plot after linear regression, where the y-intercept at zero frequency denotes the frequency-dependent hysteresis loss part, while the linearly increasing values represent the contribution of eddy current loss as a function of frequency (value of the slope). For 0.5 T, MS-N_2_ exhibited a *P_eddy_* (*F*) contribution of ~0.000002*F* W/kg and a *P_hys_* (*F*) contribution of ~0.0032*F* W/kg; MS-He showed a *P_eddy_* (*F*) of ~0.000003*F* W/kg and a *P_hys_* (*F*) of ~0.0011*F* W/kg, and Metglas 2605SA1 depicted a *P_eddy_* (*F*) part of ~0.000003*F* W/kg and a *P_hys_* (*F*) of ~0.0012*F* W/kg. At 1.0 T amplitude, the loss components of eddy current and hysteresis corresponded to ~0.000007*F* W/kg and ~0.0085*F* W/kg for MS-N_2_, ~0.00001*F* W/kg and ~0.0042*F* W/kg for MS-He, and ~0.00001*F* W/kg and ~0.0041*F* W/kg for the reference material, respectively. Thus, it is evident that the hysteresis losses are substantially higher than the eddy current losses by spans of 2 to 3 orders of magnitude. The energy lost to hysteresis per cycle per unit volume is proportional to the area enclosed by the quasi-static hysteresis loop [74], and the excess loss primarily arises from the domain wall dynamics [1,72]. The very low coercive fields of MS-He and Metglas 2605SA1 gave rise to substantially narrower hysteresis loops than MS-N_2_, as shown in Figure 5b, and consequently to much lower total losses at both 0.5 T and 1.0 T, which is also consistent with the values evaluated from the loss segmentation. The quasi-static hysteresis loops of MS-He and the commercial reference are nearly identical for *H* < 100 A/m. Consequently, both MS-He and Metglas 2605SA1 showed negligible differences in their hysteresis part, and at very low frequencies of 50 and 100 Hz, the total losses of these two materials exhibited minimal deviations at both *J*~0.5 T and *J*~1.0 T (Figure 5c,d). However, at higher frequencies, MS-He showed even lower losses than Metglas 2605SA1 at *J*~0.5 T, while at *J*~1.0 T, the trend reversed. These differences are plausibly attributable to variations in their excess loss contributions under varying AC excitations. Excess losses tend to increase with higher saturation magnetostriction, suggesting that magnetostriction significantly influences domain wall mobility [75]. In addition, at higher frequencies, the coherence of domain wall movement could be affected, which can also lead to elevated excess losses [75,76]. Therefore, an in-depth study of the excess loss mechanism and its influencing factors could further interpret the anomalies observed for the core losses of MS-He and Metglas 2605 SA1 at 0.5 T and 1.0 T.

## 5. Conclusions

The investigations carried out in this study led to the following conclusions:

- Helium gas showed excellent efficiency as a quenching atmosphere to successfully produce fully amorphous melt-spun ribbons of Fe80B13Si7, whereas under identical process parameters, quenching in nitrogen showed a higher propensity toward devitrification and a non-uniform structure across the ribbon thickness.
- The optimal annealing window for achieving superior magnetic softness was identified as approximately 15 to 20 K below the respective Curie temperatures of the samples.
- Helium-quenched samples exhibited an extremely low average coercivity of ~1.3 A/m closely approaching that of the reference material (*H_c_*~0.6 A/m) and a maximum permeability of 28.4 ± 1.3 (×10^3^) comparable to the Metglas 2605SA1 (33.3 ± 4.8 (×10^3^)).
- Both helium- and nitrogen-quenched soft magnetic ribbons displayed similar saturation polarization values (1.60–1.64 T), distinctly higher than the commercial reference material (1.54–1.56 T).
- Quenching in helium gas also resulted in an excellent surface quality (*S_a_*~0.26 μm), yielding a lower arithmetic mean areal roughness than the reference material (~0.34 μm).
- Helium-quenched ribbons exhibited substantially lower core losses than nitrogen-quenched ribbons and even outperformed Metglas 2605SA1 in efficiency at a magnetic polarization of *J*~0.5 T.

Overall, helium as a quenching atmosphere showed strong potential for promoting amorphous structure formation, thereby enabling excellent soft magnetic properties and very low energy losses. Although the cooling rates were not measured in the present study, their direct quantitative evaluation under different processing atmospheres would be valuable in future work to establish a more detailed understanding of the underlying mechanisms. Owing to their properties, performance, and tiny surface undulations, Fe80B13Si7 ribbons produced in helium may deliver enhanced stacking efficiency and functionality for transformer core applications, although dedicated lamination-factor measurements have to be carried out in future work to confirm this potential. Industrial implementation of helium would require strategies to minimize helium consumption, such as closed-loop gas recovery and recycling. Future studies should also demonstrate that improvements in ribbon quality and magnetic performance provide sufficient added value at the transformer core level to justify the additional cost relative to nitrogen. In addition, whether alloys with slightly elevated Fe content (about 81–85 at%) exhibit improved GFA in helium gas, thereby attaining higher *J_s_* and enhanced magnetic behavior, has yet to be examined.

## Figures and Tables

**Table 1:** Essential process parameters during melt-spinning and the physical properties of the fabricated ribbons and the reference specimen.

Sample Name/ Composition, at%	Quenching Atmosphere	Overpressure, (Δ*P*), Mbar	Wheel Velocity, (*U*), m/s	Melt-Ejection Temperature, °C	Thickness, µm	Width, mm	Density, g/cm^3^
MS-He/Fe80B13Si7	Helium	~240	~20	1240 ± 5	21.25 ± 0.33	9 ± 0.2	7.18 ± 0.04
MS-N_2_/Fe80B13Si7	Nitrogen				23.97 ± 0.88		7.22 ± 0.09
Metglas 2605SA1/Fe80B11Si9 [41]	-	-	-	-	~23 [42]	~210	7.18 [42]

**Table 2:** Key magnetic and physical properties of the in-house fabricated ribbons and Metglas 2605SA1.

Sample Name	Coercivity (*H_c_*), A/m		Maximum Permeability (*μ_max_*), a.u.		Specific Electrical Resistivity (*ρ*), μΩ·cm		Arithmetic Mean Height of the Surface (*S_a_*), μm	Saturation Polarization (*J_s_*), T
	As-Spun	Heat-Treated	As-Spun	Heat-Treated	As-Spun	Heat-Treated	As-Spun	Heat-Treated
MS-He	2.52 ± 0.83	1.26 ± 0.37	8.3 × 10^3^	28.4 ± 1.3 (×10^3^)	143.1± 1.6	141.6 ± 2.1	0.26 ± 0.03	1.63 ± 0.01
MS-N_2_	18.95 ± 3.13	14.06 ± 0.54	6.4 × 10^3^	8.9 ± 0.3 (×10^3^)	140.3 ± 2.8	139.4 ± 2.1	0.54 ± 0.06	1.61 ± 0.01
Metglas 2605SA1	4.64 ± 0.48	0.59 ± 0.16	6.0 × 10^3^	33.3 ± 4.8 (×10^3^)	138.9 ± 1.6	139.6 ± 2.2	0.34 ± 0.02	1.55 ± 0.01

## Data Availability

The original contributions presented in this study are included in the article. Further inquiries can be directed to the corresponding authors.
